# EEG Investigations of Duration Discrimination: The Intermodal Effect Is Induced by an Attentional Bias

**DOI:** 10.1371/journal.pone.0074073

**Published:** 2013-08-23

**Authors:** Emilie Gontier, Emi Hasuo, Takako Mitsudo, Simon Grondin

**Affiliations:** 1 Laboratoire de Recherche en Psychologie de la Perception, Université Laval, Québec, Québec, Canada; 2 Faculty of Medical Sciences, Kyushu University/JSPS, Fukuoka, Japan; 3 Faculty of Information Science and Electrical Engineering, Kyushu University, Fukuoka, Japan; National University of Singapore, Singapore

## Abstract

Previous studies indicated that empty time intervals are better discriminated in the auditory than in the visual modality, and when delimited by signals delivered from the same (intramodal intervals) rather than from different sensory modalities (intermodal intervals). The present electrophysiological study was conducted to determine the mechanisms which modulated the performances in inter- and intramodal conditions. Participants were asked to categorise as short or long empty intervals marked by auditory (A) and/or visual (V) signals (intramodal intervals: AA, VV; intermodal intervals: AV, VA). Behavioural data revealed that the performances were higher for the AA intervals than for the three other intervals and lower for inter- compared to intramodal intervals. Electrophysiological results indicated that the CNV amplitude recorded at fronto-central electrodes increased significantly until the end of the presentation of the long intervals in the AA conditions, while no significant change in the time course of this component was observed for the other three modalities of presentation. They also indicated that the N1 and P2 amplitudes recorded after the presentation of the signals which delimited the beginning of the intervals were higher for the inter- (AV/VA) compared to the intramodal intervals (AA/VV). The time course of the CNV revealed that the high performances observed with AA intervals would be related to the effectiveness of the neural mechanisms underlying the processing of the ongoing interval. The greater amplitude of the N1 and P2 components during the intermodal intervals suggests that the weak performances observed in these conditions would be caused by an attentional bias induced by the cognitive load and the necessity to switch between modalities.

## Introduction

Understanding the neural and cognitive mechanisms underlying time perception is a fine challenge. Indeed, even though there are no sensory organs dedicated to time perception [1], it is possible to process durations within various sensory modalities (auditory, visual, tactile). We are thus able to perform a comparison between the duration of visual and auditory stimuli [2] or to reproduce by touch on a keyboard the duration of stimuli presented in the visual modality [3]. This capability to integrate multisensory information over time, in a time scale of milliseconds to seconds, is central for developing a coherent perception of the world and to adapt behaviour to environmental changes. However, although time perception seems to transcend, at first glance, the sensory modality of the temporal information, different data lead to moderate this assumption. Indeed, some behavioural studies indicated that the ability to process durations is influenced by the sensory input. It has been shown that temporal intervals are judged as longer when marked by auditory rather than by visual stimuli [4]. In addition, different data have revealed that sensitivity to time is better (lower discrimination threshold, less variability) in the auditory rather than visual modality, a finding that applies to both filled and empty intervals [5–7]. This auditory dominance for temporal processing has also been observed with sequences of flashes or tones and with auditory and visual rhythms [2,8].

In agreement with physiological data showing an overlap of the neural systems underpinning explicit timing [9], the superiority of the auditory over the visual system on time processing has been mainly explained within the framework of a centralized clock. In particular, the Scalar Expectancy Theory (SET) proposed by Gibbon, Church, and Meck in 1984 [10] has guided numerous investigations and served as a reference for studying time perception in animals and in humans. This model posits that time judgments rely on an internal clock, described as a pacemaker-accumulator device. According to SET, the pacemaker emits pulses at a constant mean rate which are transferred into an accumulator. Transfer of pulses is accomplished by means of a switch, which closes and opens respectively at the onset and the offset of a timed event. This switch is reported to be under attentional control. Reduced attention allocated to time could increase the latency of the switch closure or cause the switch to fluctuate between the closed and the open state [11]. The value contained in the accumulator is transmitted to working memory before being compared to the representation of a standard duration, stored in long-term memory. The judgment of the elapsed time depends on the relationship between these two values.

Consistent with the single-model hypothesis, it has been proposed that these different levels of discrimination [5], as well as perceived duration [12], would be related to the variability induced by the sensory signals themselves. Some authors have proposed that the lengthening of the subjective duration for auditory stimuli could be related to the speed of the pacemaker (faster for auditory than visual). It has also been suggested that the higher performances observed in the auditory modality would be linked to a lower variability in the latency of the switch closure or opening and to the efficiency of auditory signals to maintain the switch in the closed mode (for review, see 4,13). However, for some authors, these interpretations make it difficult to explain the results collected in tasks using intermodal intervals, that is, intervals delimited by signals of different sensory modalities. Suppose we have two stimuli, one auditory and one visual, with unequal levels of efficiencies, i.e. inducing more or less variability for marking an interval to be discriminated. One would predict that using the most efficient (auditory) sensory stimulus to delimit the beginning and the end of the interval will lead to better performance than using two visual signals, and using an auditory signal followed by a visual signal, or the reversed (visual followed by auditory), should induce intermediate levels of performance. However, various data contradict with this prediction: Using visual-auditory or auditory-visual signals for delimiting respectively the onset or the offset of an interval severely impairs performance. For brief durations, intramodal intervals (delimited by the same sensory modality) are always much easier to discriminate than intermodal intervals (delimited by distinct sensory modalities) [14–17]. These results have therefore led some authors to question the validity of centralized clock notion. For instance, Grondin and Rousseau [15] have proposed that there could be two types of mechanisms for processing time intervals: one specific (to any given sensory modality) and one aspecific (which would act in intermodal conditions). Somewhat along the same line, Mauk and Buonomano [18] have suggested that the decline of the performance observed in intermodal conditions could be related to the fact that the stimulus features that delimit the interval arrive at different timers and would constitute an argument in favour of intrinsic timing models [19–22] (for review, see 23) and neurophysiological data suggesting a role of modality-specific areas in time processing ( [24–26]).

However, as pointed out by the authors themselves [18], no argument can rule out, at this time, the idea that the poor performance in intermodal timing would be related to the cognitive load generated by this kind of processing. Indeed, even though behavioural studies offer the possibility to know how experimental factors affect the subjects’ responses, they cannot determine with high precision the different processing stages that have led to these responses.

Electroencephalography constitutes a technique of choice in the study of time processing since it can record precisely the change in brain activity over time from the beginning of the stimuli until the judgment is made. Especially, some components of event related potentials (ERPs) could help us to better understand the differences in performance observed as a function of the sensory modalities involved in the presentation of intervals to be discriminated. As such, the Contingent Negative Variation (CNV) is an index of choice in the understanding of timing mechanisms. Since its discovery by Walter, Cooper, Aldridge, McCallum and Winter in 1964 [27], numerous studies have revealed relationships between the CNV characteristics and the processing stages of time intervals in the sub- and supra-second ranges [28]. It has been found that its amplitude and resolution were correlated with temporal accuracy [29–33]. Furthermore, various studies have suggested that its amplitude would be an index of subjective duration, the CNV being larger when durations were judged longer rather than shorter [34,35]. The evolution of the characteristics of the CNV during timing tasks was initially interpreted, in the perspective of SET, as a reflection of the accumulation of pulses, which is probably based on the activity of climbing neurons [36]. This first interpretation has recently been challenged by Kononowicz and Van Rijn [37] who failed to replicate the performance-dependent variations of the CNV observed previously. In addition, they found a habituation effect, that is, a time-on-task decreasing effect in the CNV amplitude, which would hardly be compatible with the cumulative original interpretation. As a consequence, two alternative explanations have been proposed. The first is that the increase of the CNV amplitude over time could reflect oscillatory processes proposed in the Striatal Beat Frequency model of timing [38]. The second is that this component could reflect the unfolding of time in a more indirect way, like the anticipation of an upcoming and relevant event or decision-making processes [39,40]. This latter proposal is in line with the results of various investigations showing that the CNV amplitude stopped rising at the end of a standard duration, even when the test duration went beyond, suggesting that its peak amplitude would reflect the representation of a previously memorized duration [41,42]. The relationship between the CNV peak and an expected duration has subsequently been replicated in implicit and passive timing tasks ( [43,44]), suggesting that this effect represents a robust phenomenon in the timing literature. In other words, although the CNV amplitude is subject to contradictory interpretations, many data confirm that the characteristics of this component, particularly its peak amplitude, are markers of timing mechanisms.

Moreover, although these components have been little studied within the framework of temporal processing (but see e.g. 34,45), the N1 and P2 components are also important clues in understanding the mechanisms underlying the intra- and intermodal time perception. Indeed, while these transient evoked components reflect the processing of physical properties of stimuli, it has also been shown that their characteristics vary depending on the attention allocated to sensory signals (for reviews, see 46,47).

Given the high temporal resolution of electroencephalography and the contribution of the ERPs studies to the understanding of the neural mechanisms underlying timing perception, it is surprising that this technique has been so little used to study the influence of the sensory modality on time processing (but, see e.g 48–50). Moreover, while the majority of electrophysiological studies have evaluated the influence of sensory modality on implicit timing, few studies have been undertaken to address this issue in the context of an explicit temporal task. Among these studies, that of N’Diaye, Ragot, Garnero, and Pouthas [49], using EEG and MEG co-recordings, has attempted to determine the evolution of the cortical activity recorded in explicit timing tasks involving the processing of filled auditory or visual interval, that is, intramodal intervals. However, no electrophysiological studies have been conducted to determine the mechanisms involved when participants have to process intermodal intervals. A corpus of electrophysiological data could provide crucial information to the understanding of the elusive intermodal effect. Indeed, although this technique does not allow us to determine exactly the brain regions involved in the temporal processing when modality issues are investigated and, therefore, to test the existence of multiple timers, it can, however, inform us about the cognitive mechanisms involved during an intermodal processing. Processing of intermodal intervals could differ from that of intramodal intervals in certain ways. For example, a shift of attention from one modality to another is required for perceiving durations marked by intermodal stimuli [7]. This could affect the accumulation of pulses and explain the impairment of performances. As a consequence, it has been judged relevant to conduct an electrophysiological experiment in order to determine whether cognitive processes could account for the different levels of performance observed between intra- and intermodal intervals. Furthermore, while a comparison of the evoked potentials has been performed between auditory and visual modalities for filled intervals [49], to our knowledge, no electrophysiological study has been conducted to determine the influence of these two sensory modalities on the explicit timing of empty intervals. Using empty intervals could help overcome some limits of the electroencephalography. Indeed, besides the transient evoked components (e.g., N1-P2 complex), it is now well established that the presentation of a continuous sensory stimulus induces the development of sustained evoked potentials ( [51,52]). These negative potentials can be observed on the same electrodes as the transient sensory response and appear approximately 100 ms after the onset of the stimulation. As a consequence, they may overlap with the other slow waves, making the distinction between sensory- and timing-related activities difficult. Since these components appear for sensory stimuli lasting few hundred milliseconds, the use of short signals to delineate the intervals could be a way to reduce this confounding factor.

In this context, the present study was conducted to determine, using ERPs, the origins of differences in performance observed in inter- and intramodal conditions. Specifically, this investigation had two main objectives. The first was to evaluate, through the evolution of the CNV characteristics, the influence of the sensory modality on the processing of empty intervals, delimited by signals of short durations. The second was to define, through the study of N1 and P2 components, whether the difficulty to discriminate intermodal intervals results from a disturbance of the attentional mechanisms. To access this and in order to compare our results with those obtained previously in the literature, the electrophysiological patterns of activities and the behavioural responses have been studied in a temporal task similar to that used by Grondin and Rousseau [15], which was based on the single stimulus method [53].

## Methods

### Participants

Sixteen participants (4 men, 12 women with a mean age of 25 years; range: 19-34 years), recruited among the employees or students at Laval University, took part in this experiment. They were right-handed according to the 32-item version of the Waterloo Handedness Questionnaire [54], had no history of neurologic or neuropsychiatric disorders, no hearing deficits and normal or corrected-to-normal visual acuity. They were informed of the conditions of the study and signed a written consent, allowing them to stop the experiment at any time. The experiment was approved by the Ethics Committee of Laval University and each participant received $30 CAN for his/her contribution to the study.

### Stimuli

Each participant was seated in a comfortable chair in light- and sound-attenuated room, at a viewing distance of 60 cm from a 14-inch CRT monitor (refresh rate: 60 Hz) connected to a computer. Stimuli were displayed using E-prime 1.2 (©Psychology Software Tools), on a black background. An empty black circle (diameter: 1 cm) with white surrounds (1 mm, contrast 100%) displayed at the center of the screen was used as fixation point (0.95° of visual angle). The visual stimulus was a 33-ms flash (filled white circle on black background) with the same size and position as the fixation point. The auditory stimulus was a 33-ms sound (1kHz pure tone burst) including a rise and a fall time of 5 ms that were raised-cosine shaped. Sounds were presented binaurally at 80dB SPL via speakers placed on each side of the computer screen.

### Experimental procedure

The single stimulus method was used for this experiment, i.e., only a single time interval was presented per trial to participants. The interval to be discriminated was a silent duration (empty duration) between two stimuli, auditory (A) or visual (V). This empty interval could be short (450 ms) or long (550 ms).

**Figure 1:**
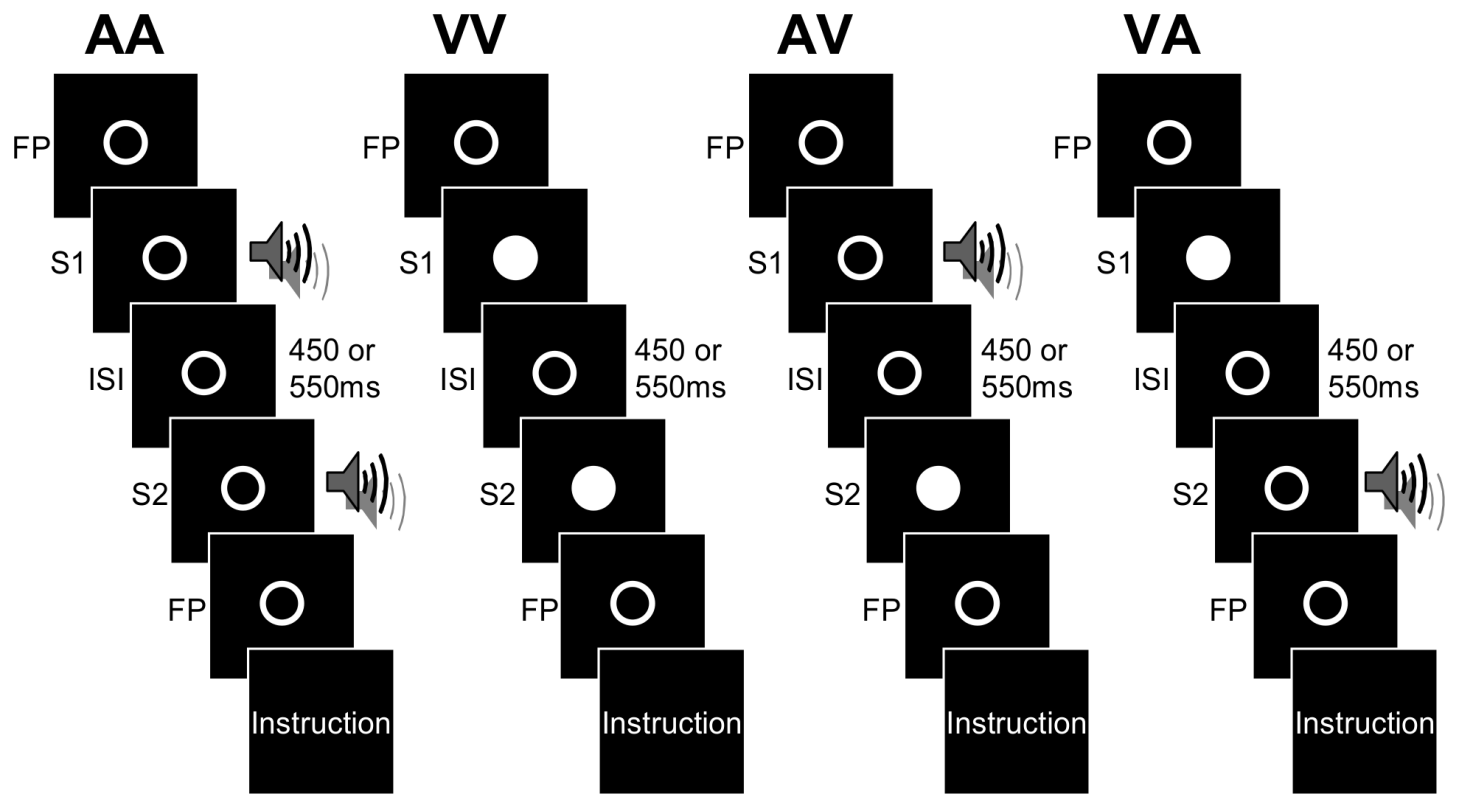
Schematic representation of the experimental paradigm. The task was to discriminate empty intervals delimited by auditory (A) and/or visual (V) signals. In two sessions, the intervals were defined by two signals of the same modality (intramodal intervals: AA or VV) while in the other two, the signals were delivered from different modalities (intermodal intervals: AV or VA). Each trial began with a fixation point (FP), followed by the presentation of an empty interval (ISI= 450 or 550 ms) bounded by auditory and/or visual stimuli (33 ms each) (S1 and S2 = Stimuli 1 and 2). Then, after the presentation of a fixation point (FP), a visual instruction asked the participants to indicate whether the empty interval corresponded to the short or long interval by pressing respectively “1” or “2” on a Serial Response Box.

The experiment consisted of four sessions corresponding to four modalities of presentation (Figure 1). In two sessions, the intervals were defined by two signals of the same modality (intramodal intervals: AA or VV) while in the other two, the signals marking the onset and the offset of the interval were delivered from different modalities (intermodal intervals: AV or VA). The order of the four sessions was counterbalanced over participants and the subjects knew in advance what stimuli would delimit the interval. Each session was divided into four experimental blocks of 50 trials (25 short and 25 long intervals presented in a randomized order within each block) and was preceded by a practice block of 10 trials. For each trial, a fixation point was displayed at the center of the screen for approximately 2750 ms, (randomized between 2500 and 3000 ms), followed by the presentation of one of the two empty intervals (450 or 550 ms) bounded by auditory or visual stimuli (33 ms each). The offset of the stimulus marking the end of the interval was followed by a fixation point lasting about 1000 ms to avoid the contamination of the evoked potentials by activity of the motor response [55]. Then, a visual instruction appeared on the screen, asking the participant to indicate whether the interval between the offset of the first stimulus and the onset of the second stimulus corresponded to the short or long duration by pressing respectively “1” or “2” with their dominant (right) hand on the Serial Response Box (SRBox-eprime). In the practice blocks, participants received feedback after each response indicating whether the presented interval was short or long. In the experimental blocks, there was no feedback and the response determined the beginning of the next trial.

### ERPs recording

Scalp voltages were continuously recorded using a 32-channel Geodesic Sensor Net™, connected to a DC-coupled 32-channel, high input impedance amplifier (NetAmps 300 TM, Electrical Geodesics, Inc., Eugene, OR). The net was adjusted so that the electrodes were correctly located according to the 10/20 system. Stimuli were presented with E-Prime software which sends coded trigger pulses to the EEG system to mark stimuli onset. EEG signals were recorded relative to a vertex reference electrode (Cz). Amplified analog voltages were digitized at 500 Hz and impedances were kept below 50 kΩ, as recommended for this high-input impedance amplifier [56]. Electrooculogram (EOG) was monitored using electrodes placed below each eye and the participants were warned about artifacts and encouraged to minimize, as much as possible, eyes blinking, muscular and ocular movements [57]. The EEG data were analyzed offline using Net Station 4.3 software (Electrical Geodesic Inc.) and digitally low-pass filtered at 30Hz. The continuous EEG was segmented into 1800-ms epoch starting 200 ms prior to S1 onset. The 200-ms pre-stimulus served as the baseline. After the segmentation, artifact detection was conducted with Net Station’s artifact detection tool, which automatically detected eye blinks, eye movements and marked bad channels in the input file. A channel with more than 100 μV between its minimum and maximum amplitude values for a given segment was identified as a bad channel for that segment. A channel was marked as bad throughout the entire recording if it was marked bad for more than 10% of the segments. Segments with eye-blink (> ± 100 μV), eye-movement (> ± 55 μV) or with more than 5 bad channels were excluded from further analyses. In the remaining segments, signal from rejected electrodes was replaced using the “bad channel replacement” algorithm in Net Station 4.3, which interpolates the signal of a bad channel from the signal of remaining channels using spherical splines. A baseline correction was applied and the average waveforms were re-referenced to averaged mastoids.

### Statistical Analyses

For behavioural data, an analysis of variance (ANOVA) was performed on the percentage of correct responses, with 4 (Modalities) × 2 (Durations) as within-subject variables. For electrophysiological data, analyses were conducted on the CNV component recorded over the fronto-central and parietal electrodes showing the largest amplitude, i.e., the fronto-central electrodes F3, F4, Fz, FCz, C3, C4 and Cz (corresponding to electrodes 3, 4, 17, 28, 5, 6 and REF in the geodesic sensor net) and the parietal electrodes P3, P4 and Pz (corresponding to electrodes 7, 8 and 19 in the geodesic sensor net). Given that the pattern of activity was qualitatively different in the fronto-central vs. parietal sites, and in conformity with temporal studies suggesting that these areas would be involved in different processing [30,32], separate analyses have been conducted for fronto-central and parietal electrodes. A first ANOVA was performed on the mean CNV amplitude calculated between 250–450 ms (short duration) and 250-550 ms (long duration) over fronto-central (4 Modalities × 2 Durations × 7 Electrodes) and parietal electrodes (4 Modalities × 2 Durations × 3 Electrodes). Then, in agreement with the work of Pfeuty et al. [42], a second analysis was conducted on the CNV time course on the left (F3/C3), right (F4/C4) and medial (Fz/FCz/Cz) fronto-central electrodes grouping and on the left (P3), right (P4) and medial (Pz) parietal electrodes. To determine whether there was an increase in amplitude of the CNV over time, the mean CNV amplitude was measured over successive temporal windows of 48 ms duration each. For the long duration, the component was divided into 6 temporal windows (tw1: 250-298, tw2: 300-348, tw3: 350-398, tw4, 400-448, tw5: 450-498, tw6: 500-548); for the short duration, the component was divided into 4 temporal windows, which corresponded to the first 4 windows used for the long duration. An analysis of variance was then carried out on the mean CNV amplitude calculated over these successive temporal windows at lateral fronto-central electrode grouping (F3/C3 and F4/C4) and at lateral parietal electrodes (P3 and P4) for the short (4 Modalities × 4 Temporal Windows x 2 Laterality) and the long duration (4 Modalities × 6 Temporal Windows x 2 Laterality). The same analyses were also performed at medial fronto-central electrodes grouping (Fz/FCz/Cz) and medial parietal electrode (Pz) for the short (4 Modalities × 4 Temporal Windows) and the long duration (4 Modalities × 6 Temporal Windows).

Statistical analyses were also conducted on the baseline-to-peak amplitude of N1 and P2 components which appeared after the presentation of the first stimulus (S1). Since the peak latency of the visual and the auditory N1 and P2 components were not the same, different windows were used to determine the peak amplitude of each component. The auditory and visual N1 were defined as the largest negative peaks between 64-164 ms and 104-204 ms after the S1 onset, respectively. The auditory and visual P2 were defined as the maximum positive peaks between 134-234 ms and 164-264 ms after S1 onset, respectively. For these components, an analysis was conducted on midline electrodes, where the amplitude was largest. In order to have an equivalent inter-electrodes distance between each site (i.e frontal, central, parietal and occipital sites), the analysis was performed on Fz, Cz and Pz for auditory N1/P2, and Fz, Cz, Pz and Oz electrodes for visual N1/P2. To compare the activities which appeared after the presentation of a visual S1 in the intra- vs. intermodal intervals, the peak amplitude of N1 and P2 were analyzed using ANOVA with a 2 (Modalities: VV/VA) × 2 (Durations) × 4 (Electrodes) factorial design with repeated measures on each variable. Similarly, the N1 and P2 peak amplitudes which appeared after the presentation of an auditory stimulus in the intra- vs. intermodal intervals were studied using ANOVAs with a 2 (Modalities: AA/AV) × 2 (Durations) × 3 (Electrodes) factorial design with repeated measures on each variables. For each analysis, Greenhouse-Geisser corrections were appliedfor cases with 2 degrees of freedom or more and Tukey HSD was used as post hoc test.

## Results

### Behavioral data

**Figure 2:**
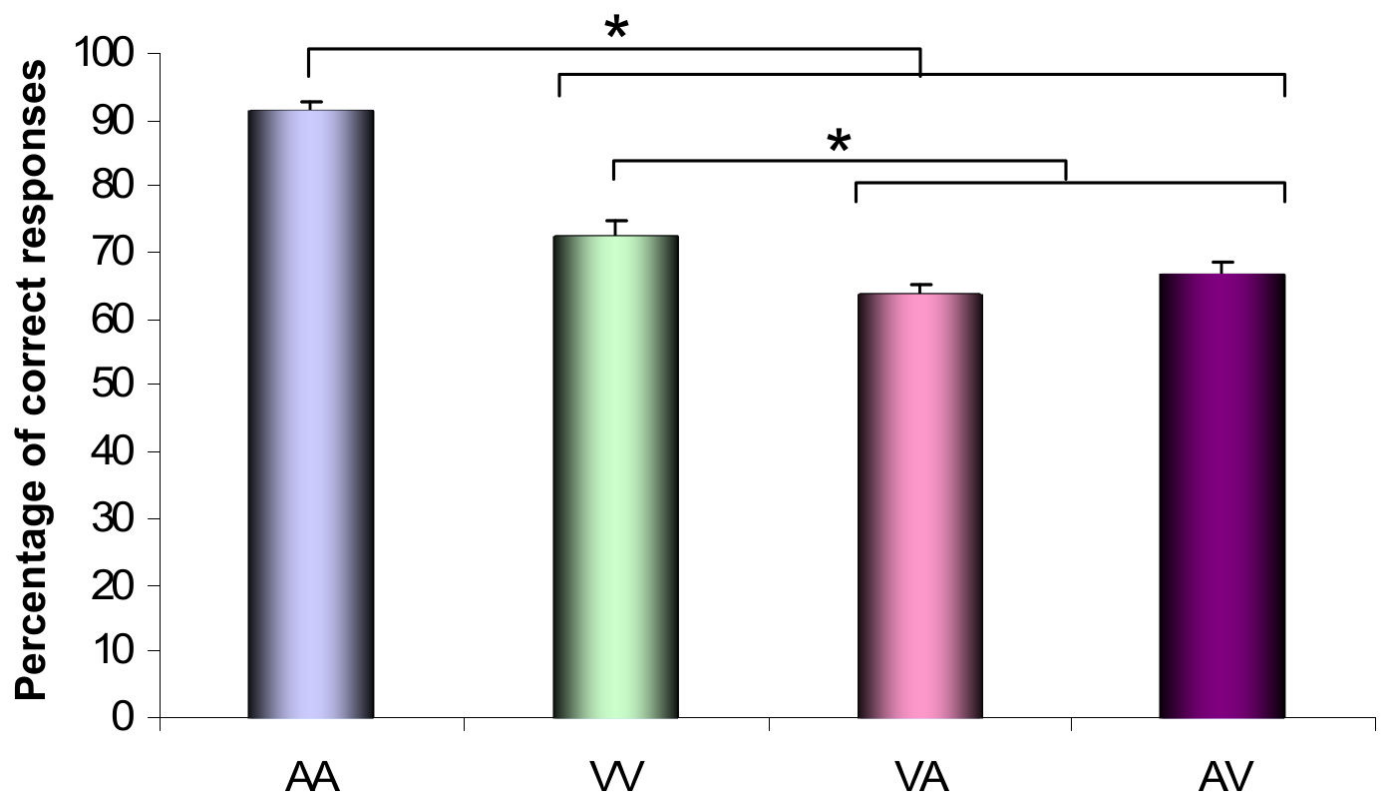
Discrimination levels. Percentage of correct responses obtained for the AA, AV, VA and VV intervals. Bars are standard errors.

The ANOVA performed on the percentage of correct responses revealed main effects of Modalities (*F*[3,45] = 96.34; *p* < .001; η_*p*_^2^= .86) and Durations (*F*[1,15] = 5.71; *p* < .05; η_*p*_^2^= .28), the latter indicating that the accuracy was higher for the short intervals than for the long intervals. As illustrated in Figure 2, Tukey test conducted on the effect of Modalities revealed better performances when subjects had to discriminate intervals delimited by two auditory stimuli (AA) compared to the three other modalities of presentation (*p* < .001). It also indicated that the accuracy was significantly higher for VV than for intermodal intervals (*p* < .05).

### ERPs data

**Figure 3:**
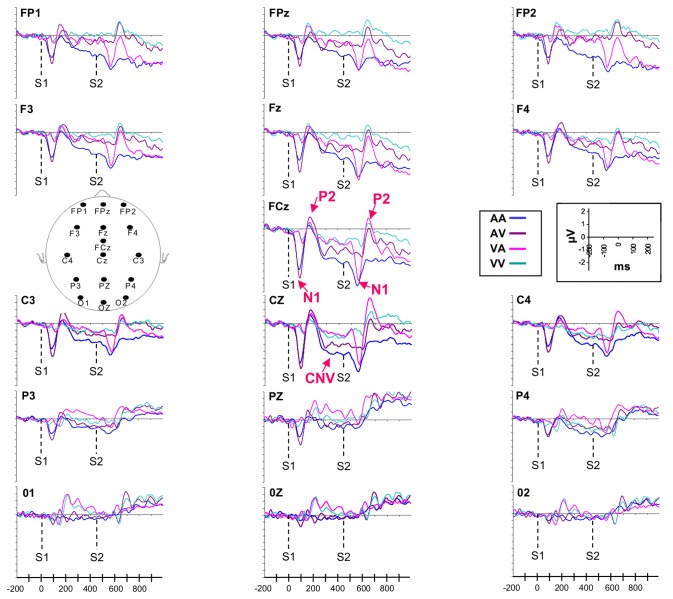
Electrophysiological activity recorded during the short interval. Data collected at prefrontal (FP1, FP2, FPz), fronto-central (F3, F4, Fz, FCz, C3, C4, Cz), parietal (P3, P4, Pz) and occipital electrodes (O1, O2, Oz) are presented.

**Figure 4:**
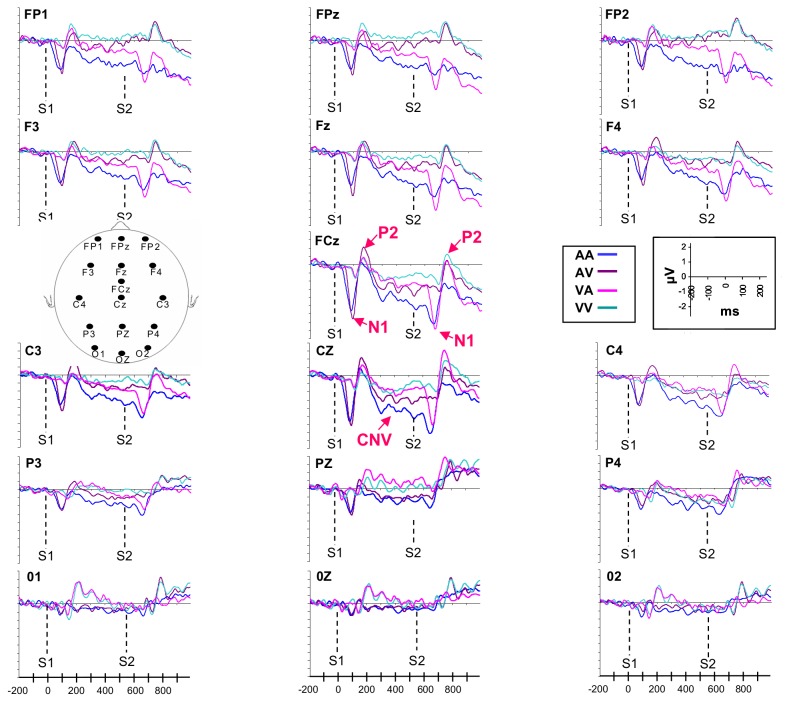
Electrophysiological activity recorded during the long interval. Data collected at prefrontal (FP1, FP2, FPz), fronto-central (F3, F4, Fz, FCz, C3, C4, Cz), parietal (P3, P4, Pz) and occipital electrodes (O1, O2, Oz) are presented.

As shown in Figures 3 and 4, after the presentation of the first stimulus (S1) which marked the beginning of the interval to be discriminated, an early negative component N1, which peaked in amplitude respectively at around 100 ms (after the auditory signal) and 150ms (after the visual signal), and a following positive component P2, which peaked at around 180 (after the auditory signal) and 210 ms (after the visual signal), could be observed. For both components, highest amplitudes appeared at midline frontal, central and parietal electrodes for all modalities and also at midline occipital electrodes for the VA and VV intervals. These early waves were followed by a negative component, the CNV, which developed mainly at fronto-central and parietal electrodes. The CNV resolution was then marked by the development of N1 and P2 components after the presentation of the second stimulus (S2) which represented the end of the interval. In this paper, we focus only on the characteristics of the CNV and the N1 and P2 components which appeared after S1. Indeed, it is well now established that the presentation of two consecutive stimuli of the same sensory modality leads to lower amplitude of the components induced by the presentation of the second stimulus, due to the refractory period [58]. It would be difficult to compare the intra- and intermodal conditions by analysing the N1 and P2 components for the second stimulus (S2), because it would not be possible to determine whether the difference between the intramodal and intermodal intervals, if any, would be due to attentional states related to the intermodality, or simply to the intra vs. intermodal refractory period differences.

#### Mean CNV Amplitude

**Figure 5:**
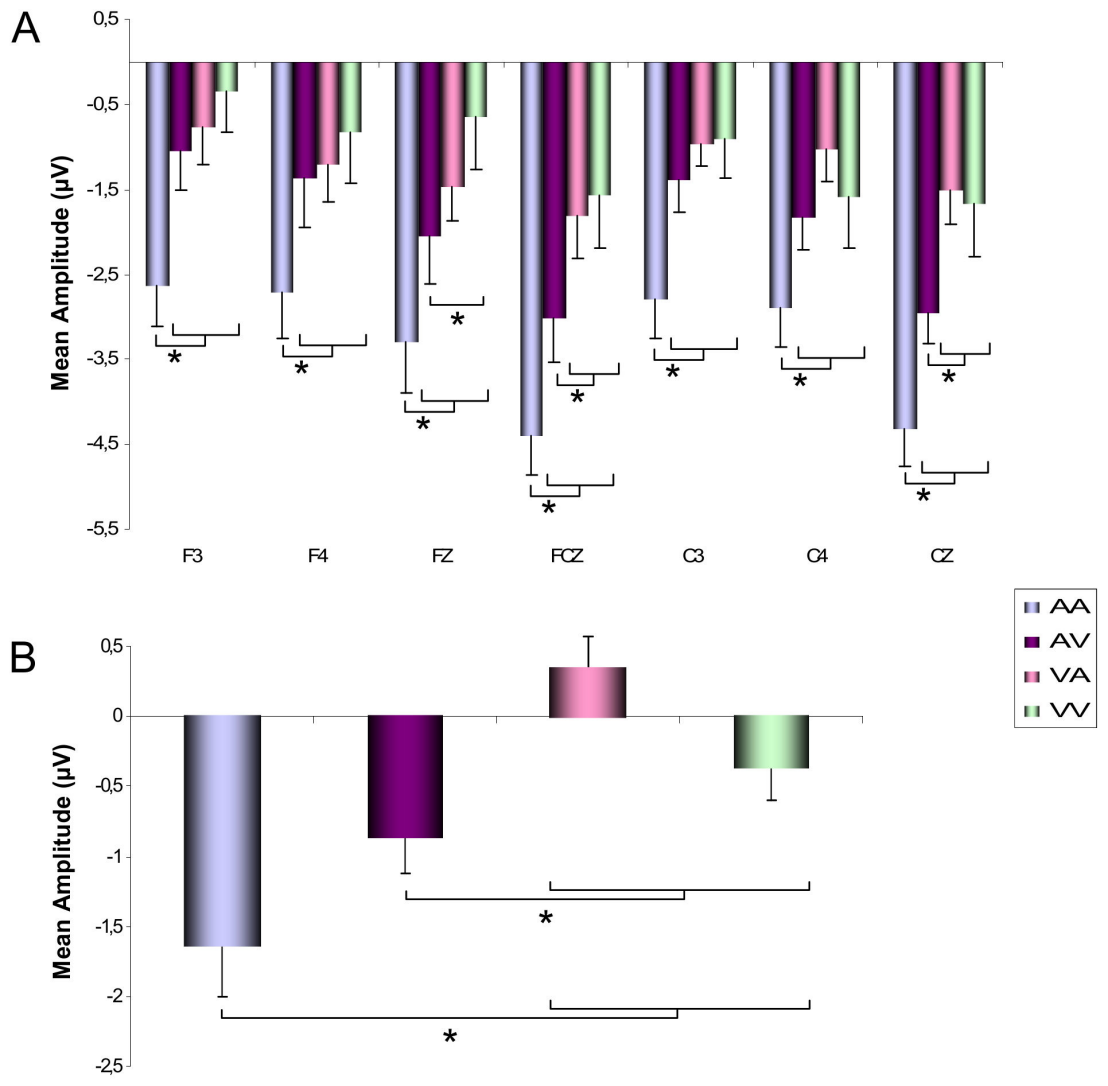
Mean CNV amplitude. Mean amplitude recorded during AA, AV, VA and AA intervals at fronto-central (F3, F4, Fz, FCz, C3, C4, Cz) (A) and parietal (P3, P4, Pz) (B) sites. Bars are standard errors.

The ANOVA conducted on the mean CNV amplitude over fronto-central electrodes revealed that the Modalities (*F*[3,45] = 11.88; *p* < .001; η_*p*_^2^= .43) and Electrodes (*F*[6,90] = 12.59; *p* < .001; η_*p*_^2^= .45) effects, as well as their interaction (*F*[18,270] = 3.27; *p* < .001; η_*p*_^2^= .17), were significant. As illustrated in Figure 5 A, the mean CNV amplitude was always larger for the AA intervals compared to the three other modalities of presentation for all fronto-central electrodes: (*p* < .001). The post hoc analysis also indicated that the amplitude was larger for the AV than for the VA intervals at FCZ and Cz electrodes (*p* < .001) and also larger compared to the VV intervals at the Fz, FCz and Cz electrodes (*p* < .001).

The same analysis conducted over parietal electrodes revealed that the mean amplitude of the component depended also on the Modalities (*F*[3,45] = 11.78; *p* < .001; η_*p*_^2^ = .44) the CNV being larger at parietal sites for the AA intervals compared to the VA and VV intervals (*p* < .001), and also larger for AV compared to VA (*p* < .01) and VV intervals (*p* < .05) (Figure 5 B). The analysis also revealed a main effect of Electrodes (*F*[2,30] = 4.92; *p* < .05; η_*p*_^2^ = .25), showing that the CNV amplitude was larger at P4 compared to PZ electrodes (*p* < .05).

#### CNV time-course

**Figure 6:**
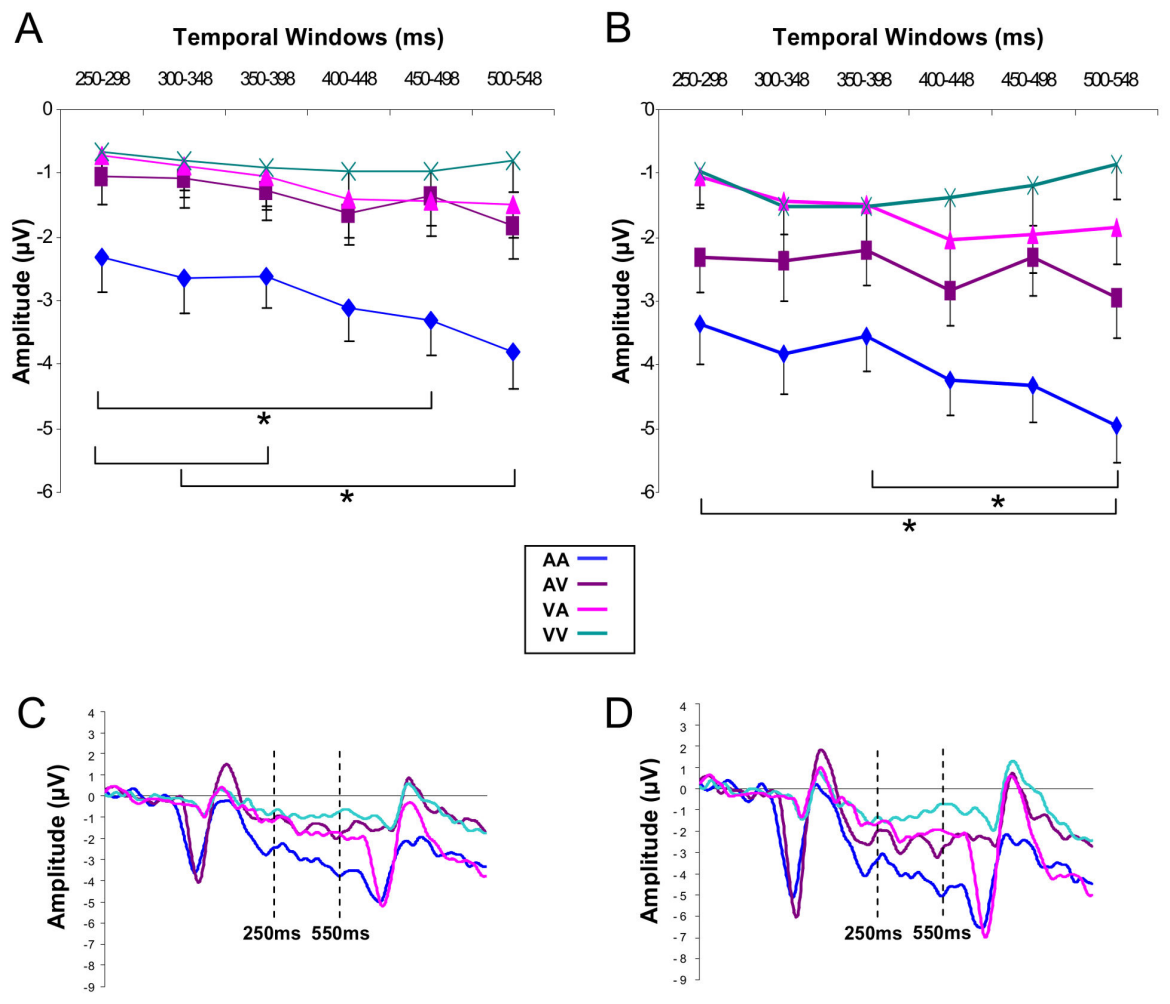
CNV time course over fronto-central sites. Mean CNV amplitude for the long interval over lateral (A) and midline (B) fronto-central electrodes for successive time windows. Bars are standard errors. ERPs elicited during the long interval and averaged over lateral (F3, F4, C3, C4) (C) and midline (Fz, FCz, Cz) (D) fronto-central electrodes.

The analysis performed over fronto-central lateral electrodes (F3-C3/ F4-C4) showed, for the long duration, significant effects of Modalities (*F*[3,45] = 7.15, *p* < .001; η_*p*_^2^ = .32) and Temporal Windows (*F*[(5, 75] = 5.07, *p* < .001; η_*p*_^2^ = .25), an significant interactions between Temporal Windows and Modalities (*F*[15,225] = 1.74, *p* < .05; η_*p*_^2^ = .11) and between Temporal Windows and Laterality (*F*[5,63] = 3.92, *p* < .01; η_*p*_^2^ = .24). For the first interaction, and as shown in Figure 6 A, the post hoc analysis revealed that the amplitude of CNV recorded for the AA interval increased between tw1 and tw5 (*p* < .01), tw1 and tw6 (*p* < .001), tw2 and tw6 (*p* < .001) and also between tw3 and tw6 (*p* < .001). In contrast, no significant differences between temporal windows were revealed for the three other intervals (VV, AV, VA). For the interaction between Temporal Windows and Laterality, the post hoc analysis indicated that the amplitude of the CNV was larger at right (F4-C4) compared to left sites (F3-C3) for tw3 (*p* < .05), tw4, tw5 and tw6 (*p* < .001). The same analysis performed on the short duration indicated an effect of Modalities (*F*[3,45] = 6.63; *p* < .001; η_*p*_^2^ = .31) showing that the amplitude of the CNV was larger for the AA than for the VA and VV intervals (*p* < .001).

The same analysis conducted over the fronto-central midline electrodes (Fz-FCz-Cz) for the long duration indicated that the effect of Modalities (*F*[3,45] = 10.92; *p* < .001; η_*p*_^2^ = .42), Temporal Windows (*F*[5,75] = 2.83; *p* < .05; η_*p*_^2^ = .15) and their interaction (*F*[15,225] = 2.34; *p* < .01; η_*p*_^2^ = .13) were significant. As for lateral electrodes, the post hoc analysis performed for each modality of presentation revealed that the amplitude of the component was modulated significantly over time only for the AA interval, the amplitude increasing between tw1 and tw6 (*p* < .001), tw3 and tw6 (*p* < .01) (Figure 6 B). For the short duration, the analysis conducted on fronto-central midline electrodes showed a main effect of Modalities (*F*[3,45] = 13.53; *p* < .001; η_*p*_^2^ = .47), indicating that the amplitude of the CNV was larger for the AA compared to the VA and VV intervals (*p* < .001) and for the AV compared to the VA and VV intervals (*p* < .05).

**Figure 7:**
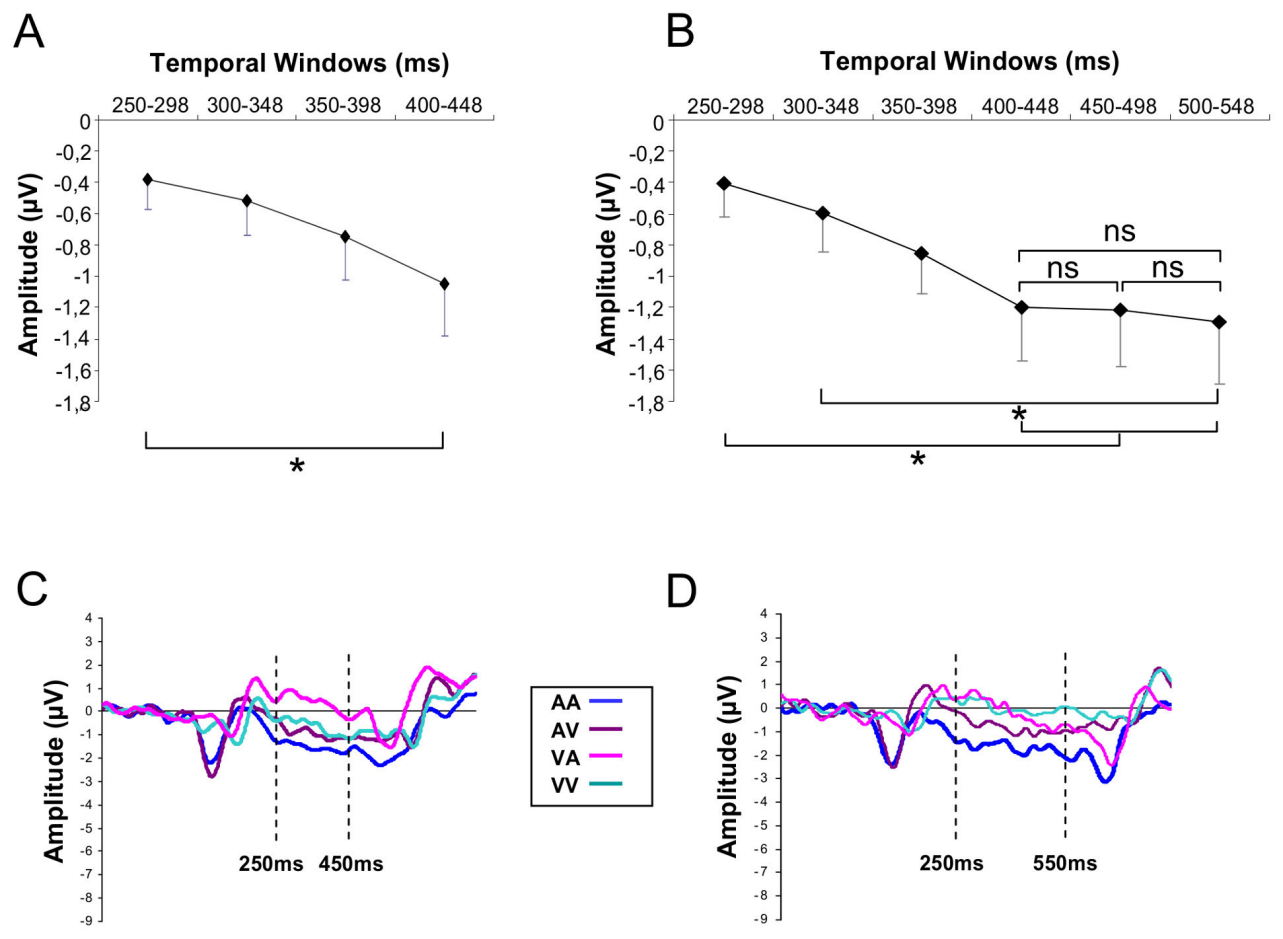
CNV time course over parietal sites. Mean CNV amplitude over lateral parietal electrodes (P3, P4) for successive time windows for the short (A) and long (B) intervals. Bars are standard errors. ERPs elicited by the short (C) and the long (D) intervals and averaged over lateral parietal electrodes (P3, P4).

The ANOVA conducted on lateral parietal electrodes (P3/P4) for the long duration revealed an effect of Modalities (*F*[3,45] = 8.31; *p* < .001; η_*p*_^2^ = .35) showing that the amplitude was larger for the AA intervals compared to the three other intervals (*p* < .05), and an effect of Temporal Windows (*F*[5,75] = 6.33; *p* < .001; η_*p*_^2^ = .29), which revealed that the amplitude of the CNV at parietal electrodes increased between tw1 and tw4 (*p* < .05), tw5 (*p* < .05), tw6 (*p* < .01) and between tw2 and tw6 (*p* < .05) (Figure 7). For the short duration, it indicated also a main effect of Modalities (*F*[3,45] = 10.08; *p* < .001; η_*p*_^2^ = .41), the AA intervals being larger than the three other intervals (*p* < .05) and an effect of Temporal Windows (*F*[3,48] = 5.21 ; *p* < .01; η_*p*_^2^ = .25), showing that the CNV amplitude increased between tw1 and tw4 (*p* < .001) (Figure 7).

The analysis conducted at PZ electrode indicated only an effect of Modalities, for the long (*F*[3,45] = 7.71; *p* < .001; η_*p*_^2^ = .34) and the short (*F*[3,45] = 6.19; *p* < .01; η_*p*_^2^ = .29) durations, the amplitude being negative for the AA and AV intervals and positive for the VV and VA intervals (*p* < .05).

#### N1 P2 peak amplitude

**Figure 8:**
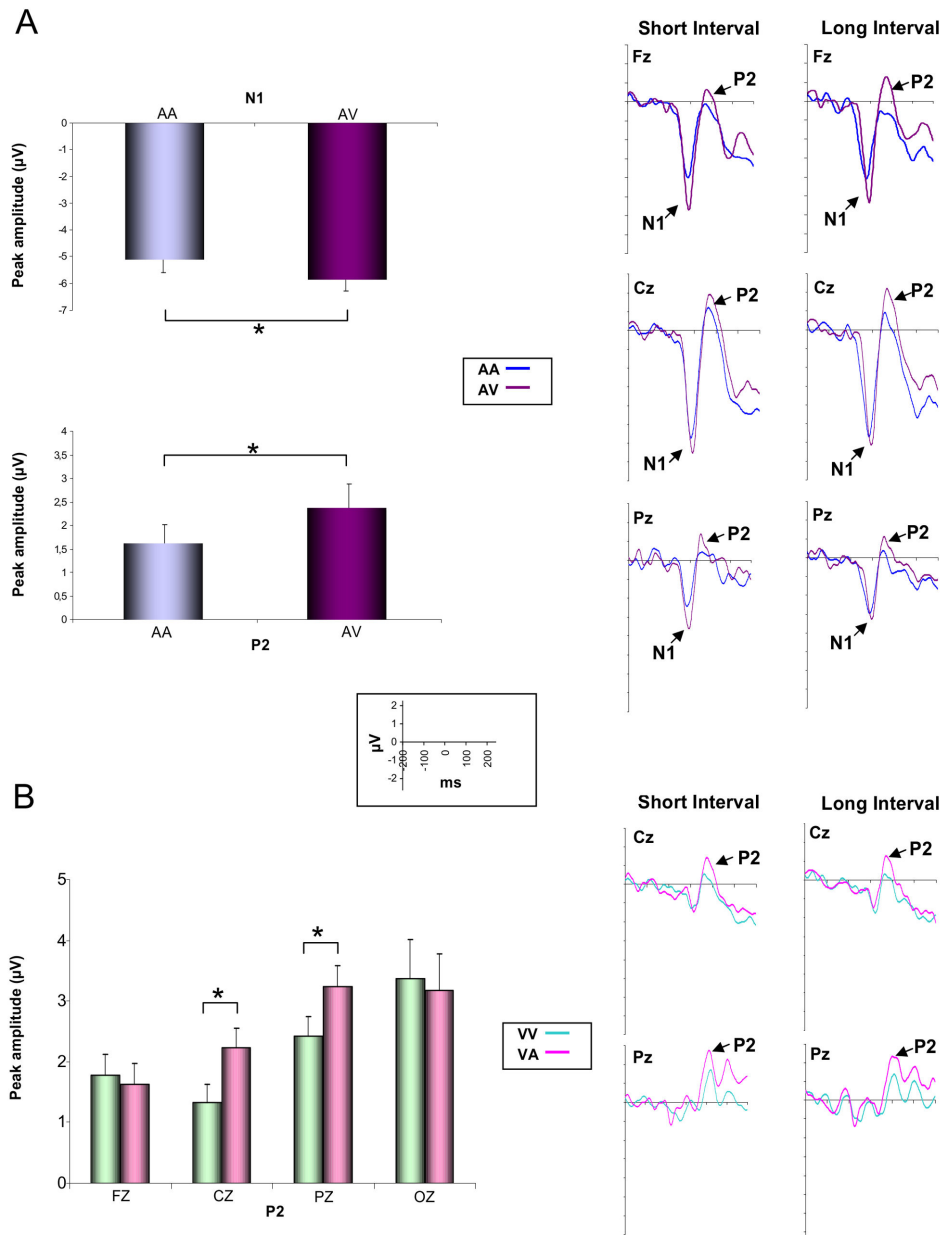
Peak amplitude of the N1 and P2 components. Peak amplitude of the N1 and P2 components and ERPs recorded at Fz, Cz and Pz after the presentation of the auditory signal which marked the beginning of the AA and AV intervals (A). Peak amplitude of the P2 component and ERPs recorded at Cz and Pz after the presentation of the visual signal which marked the beginning of the VV and VA intervals (B).

The ANOVA (2 Durations × 2 Modalities: AA/AV × 3 Electrodes) performed on the amplitude of the auditory N1 revealed a main effect of Modalities (*F*[1,15] = 4.64; *p* < .05; η_*p*_^2^ = .31), showing that the amplitude of the N1 was larger when the auditory signal delimited the beginning of the AV rather than the AA interval (Figure 8 A). It also indicated a main effect of electrodes (*F*[2,30] = 24.81; *p* < .001; η_*p*_^2^ = .63), which revealed that the N1 amplitude was larger at Cz compared to Fz (p< .05) and Pz (p< .001) electrodes and at Fz compared to Pz (p< .001) electrode. The same analysis conducted on the auditory P2 revealed also a main effect of Modalities (*F*[1,15] = 6, 17; *p* < .05; η_*p*_^2^ = .29), showing that the amplitude was higher for AV compared to AA intervals (Figure 8 A).

The ANOVA (2 Durations × 2 Modalities: VV/VA × 4 Electrodes) conducted on the visual N1 revealed no significant effect. The same analysis conducted on the P2 component indicated an interaction between Modality and Electrodes (*F*[3,45] = 3.07; *p* < .05; η_*p*_^2^ = .17), showing that the P2 component recorded at Pz and Cz electrodes was larger for VA compared to VV intervals (p< .05) (Figure 8 B).

## Discussion

### Behavioral data

Our behavioural results revealed that the percentage of correct responses was higher in the AA intervals than in the three other intervals. In addition, they indicated that the intramodal intervals were better discriminated than the intermodal intervals. These results are consistent with previous studies which have revealed that (1) visual intervals are judged with less accuracy than auditory intervals [5], [6], [7] and (2) empty intervals marked by signals of the same sensory modality are better discriminated than intervals delimited by signals of different modalities [14–17].

### ERPs data

#### Mean CNV amplitudes

The analysis of the mean CNV amplitudes collected at parietal and frontal sites revealed that the characteristics of the CNV were modulated according to the modality of the intervals to discriminate. It showed that the average amplitude of the CNV recorded at parietal site was higher for the AA and AV intervals than for the VA and VV intervals. As well, data collected at fronto-central electrodes indicated an interaction between the modalities of presentation and the electrodes location, which revealed that the amplitude of the CNV was generally larger for the AA intervals than for the other three interval types, and also higher for the AV than for the VV and VA intervals at midline electrodes (Fz, FCz, Cz and FCz, Cz, respectively). Although a parallel could be established between the larger amplitude of the CNV and the high discrimination level obtained for the AA intervals, compared to the other three intervals, the larger magnitude observed for AV compared to VV and VA intervals cannot, however, be explained in terms of processing efficiency. Indeed, the performances with the AV intervals were lower than those with VV intervals, and not significantly different from those obtained with VA intervals. Accordingly, these findings rather suggest that the mean amplitude of the CNV would depend on the physical properties of the signal marking the beginning of the intervals to discriminate. These data are thereby in agreement with previous studies which have revealed a link between the amplitude of the CNV and the sensory modality of the stimuli presented [59,60].

#### CNV time course: Parietal sites

Data collected at Pz electrode did not reveal significant differences of the CNV amplitude over time but only showed an effect of modalities on the mean amplitude of the component, a positive vs. negative polarity being observed when the beginning of the intervals was marked by a visual (VV, VA) vs. an auditory (AA, AV) signal. These data suggest, once again, the close link between the mean CNV amplitude and the sensory modality of the first stimulus. The link between the mean amplitude of the CNV and the modality of the signal delimiting the intervals could be induced by an overlap of sustained sensory evoked potentials with those related to time processing. Indeed, although we have taken the precaution of using brief sensory signals for marking intervals, we cannot exclude the hypothesis that the mean amplitude of the CNV could be modulated by the sensory inputs. On contrary, even though the mean CNV amplitude was dependent on the sensory modality of the first stimulus, our results showed that it was not the case as regard to the time course of the CNV. Indeed, data obtained at lateral parietal sites indicate a main effect of Temporal Windows, showing an increase of the CNV amplitude over time. Although the mean amplitude was generally higher for the AA than for the other three intervals at lateral sites, no interaction was found between the time course of the CNV and the modality of presentation. These results suggest that changes of the amplitude of this component over time have a similar profile for all marking conditions. Particularly, our data revealed that the amplitude of the CNV increased significantly up to 450 ms, regardless of the interval duration. In other words, the amplitude of this component increased until the end of the presentation of the short duration and stopped rising after 450 ms even when the presented interval was long.

Various data suggest that the increase in amplitude of the CNV during the presentation of the interval to discriminate would reflect the accumulation of pulses postulated in the pacemaker-accumulator model of temporal information processing [10], the oscillatory processes (CNV increases as larger groups of neurons fire simultaneously) proposed in the striatal beat frequency model [38], or expectancy mechanisms [39]. Even though our data do not allow us to plead in favour of one of these hypotheses, the activity recorded at lateral parietal electrodes suggest that the processing of the intervals would stop when the duration of the ongoing interval exceeded the shortest interval. This may reveal that the encoding of the short duration serves as standard duration, which would provide the basis for establishing temporal discrimination. Indeed, different studies have indicated that the CNV that develops during the presentation of a time interval rise until the end of the duration of a memorized standard and shows a decrease once that standard duration is reached [36,41]. Although in the present case, the task used did not involve an explicit encoding of a standard duration, our data suggest that the structure of the task may have induced an implicit encoding of the short duration as standard duration. Indeed, various data have shown that temporal expectations can be established implicitly according to the predictable temporal dynamics of the stimuli presented (for review, see 61). In this regard, Praamstra et al. [44] have demonstrated, using a serial choice reaction task that the implicit encoding of a standard duration induced a similar modulation of the characteristics of the CNV to that induced by an explicit encoding. In this study, stimuli were presented with a Stimulus Onset Asynchrony (SOA) of 1.5 s or 2 s, except the last one which was always of 1.75 s (deviant SOA). They showed that the CNV, recorded over lateral premotor areas during the deviant SOA, peaked at around 1.5 s when it was preceded by a series of short SOA. These data demonstrate that the standard duration does not need to be explicitly memorized, but can also be acquired through repeated presentation. The single stimulus method used in our study involved only the presentation of two durations, and it is likely that an implicit encoding of the short interval as the standard duration had occurred given the high number of repetitions of the intervals. The fact that the standard duration was encoded explicitly in the tasks conducted by Pfeuty et al. [36,42] and implicitly in our study may explain why the peak of activity corresponding to the standard duration was recorded at frontal electrodes in the previous studies [36,42] and at parietal electrodes in ours. However, the latency of the peak activity corresponding to the duration of a visual or tactile standard interval encoded explicitly was also identified at a parietal electrode (CPz) by Macar and Vidal [41]. Furthermore, although topographical inferences must be considered with caution, the pattern of activation that we recorded at P3 and P4 electrodes could be compatible with studies reporting the contribution of parietal cortex in explicit and implicit timing (for review, see 62; for metanalyses see 63). Especially, this is in accordance with data collected in humans as well as in monkeys showing that parietal activity increases with temporal expectations, that is, with the probability that an event occurs, whether the temporal predictions have been established incidentally (induced by the temporal regularity of the task) or deliberately [64–67].

However, if the activity collected at parietal electrodes reflects the establishment of a standard duration, our data suggest that this activity would be independent of the modalities of presentation and therefore of the ability to discriminate the intervals, suggesting that the peak amplitude recorded at parietal electrode in our study would not reflect an index of decision, as proposed by Macar and Vidal [41].

#### CNV time-course: Fronto-central sites

The time-course analysis of the CNV recorded during the long duration at lateral and midline fronto-central electrodes revealed interactions between the time windows and the modality of presentation. These interactions indicate that only the amplitude of the component collected for the AA condition increased during the presentation of the intervals; no significant difference of amplitude was observed during the presentation of the durations in the other three conditions for both midline and lateral electrodes. In addition, even though parietal activity increased only until the short duration had been reached, the amplitude of the CNV recorded at lateral and midline electrodes during the AA interval did not stop rising from 450 ms during the presentation of the long duration. Indeed, it increased significantly between 400 ms and 550 ms, i.e., until the end of the presentation of the long duration. Consequently, these results suggest that the activity recorded at fronto-central electrodes reflect the processing of the ongoing duration. For the short duration, even though the analyses revealed a modality effect on the mean amplitude of the component (larger amplitude for AA at lateral and for AA and AV at midline electrodes compared to VA and VV intervals), no effect was found for the CNV time-course. This lack of effect is not surprising given the fact that the difference in amplitude observed for the AA condition during the long duration appeared only between the first 350 ms and 500 to 550ms and that the participants did not know in advance the duration of the interval.

Furthermore, analyses indicated a lateralization effect for the long duration, larger amplitudes being observed at right compared to left frontal electrodes from 350ms until the end of the interval. These findings demonstrate the involvement of the right hemisphere in the processing of the long duration. Although in the absence of source analyses we cannot determine the cerebral localization of the activity recorded, these findings seem to be in line with studies highlighting the role played by the frontal structures in the explicit estimation of a stimulus duration that is currently unfolding in time [68–72], and may reflect the role played by the right hemisphere in the anticipatory attention of an incoming sensory event [73,74]. Especially, it has been shown in a temporal orienting task that delayed targets, compared to premature or expected ones, were accompanied by right prefrontal activations [73]. This cortical activity has also been correlated with the behavioural benefit afforded by variable foreperiods in a reaction-task paradigm, the magnitude of the recorded activity being higher for longer foreperiods, i.e., when the probability of the upcoming stimulus increased [74]. Since delayed targets involve inhibition of the prepared response at the expected time interval, it has been proposed that this activity would reflect the ability of the participants to re-orient their attention on the later time point, in other words, to update temporal expectancies throughout the time course of the trial [73]. The fact that the increase of the CNV amplitude in our task has been only observed during the presentation of the long duration converges in the direction of this hypothesis. Indeed, as revealed by the results obtained at parietal electrodes, the short duration has served as reference and represented, therefore, the expected duration for the participants. Thus, it is likely that the rising of the CNV observed in the AA condition during the long intervals reflects a re-orientation of the attention, which took place at the end of the expected duration. Given the fact that the performances were better for the AA condition as compared to the others three conditions, these results suggest that the ability of the participants to process the AA intervals could be related to the neuronal mechanisms recorded at frontal sites. The time course of these activities being dependent on the ongoing duration, these findings suggests that these activities subserve the processing of the test duration and probably reflect a built-up of attentional resources toward the offset of the current interval. This attentional process is necessary to discriminate the intervals and to perform a comparison between the expected and the test duration.

On this basis, the results obtained at fronto-central sites suggest that the better performance obtained for the AA intervals compared to the other intervals could be related to the participants’ ability to mobilize efficiently their attention on the ongoing durations when they are previously informed that the task will be to process an interval bounded by two auditory signals. These results confirm once again the notion of a dominance of audition over vision for time processing (see, e.g., 2,75) and could reflect a better efficiency of the auditory channel to capture and maintain attention during time intervals. This is also supported by the fact that the rising of the CNV during the AA condition was not observed during the AV condition even though the sensory signal delimiting the beginning of the intervals was the same. These findings are evidence that the increase in amplitude observed during the AA condition does not depend on the sensory processing as such but on the neural and cognitive mechanisms underlying the processing of the ongoing duration. The failure to observe increased amplitude in the AV and VA conditions may have been induced by an attentional switching. Indeed, in these conditions, participants had to direct their attention from one modality to another in order to capture the end of the interval. However, the fact that the CNV amplitude did not increase in the VV condition suggests that this pattern of activity cannot be solely imputable to the attentional switching but could be related to the sensory signals which delimit the intervals. These results are surprising given the fact that a temporal re-orienting of attention has been observed with visual stimuli [73,74]. As a result, two hypotheses can be proposed for the results obtained at frontal sites during the VV condition. The first, which cannot be ruled out, is that the modality of the stimuli had confounded the results. Indeed, the fact that the visual sensory inputs arise at occipital sites (and at temporal sites for auditory inputs) could explain the lack of increase in CNV amplitude observed for the VV condition at frontal sites. However, given the importance of the monitoring of the ongoing duration for the efficient processing of durations, we might have expected, if it was only a matter of sensory inputs, to find the same pattern of activation as those recorded at frontal sites for the AA condition (i.e an increase of amplitude until the end of the duration) within an another cortical location. The second hypothesis is that visual stimuli take longer time to be processed than auditory stimuli, a hypothesis consistent with data obtained with simple reaction time tasks [76]. A consequence of it would be that the intervals used in our task were too short for the development of the CNV at frontal site. Indeed, the durations used in the previous studies were longer (supra-second intervals) than those used in ours. Further studies, using longer durations and source localization are needed for testing these hypotheses.

Taken together, the results of the CNV analyses appear to confirm previous electrophysiological data suggesting a functional link between the activities recorded at frontal and parietal sites during temporal processing [29,30,49]. In the case of our task, they seem to reflect two complementary processes, with parietal activities which would be linked to temporal expectations or memorized durations, and frontal activities with attentional mechanisms necessary for the processing of the ongoing duration.

#### N1 and P2 components

Despite the fact that the study of the characteristics of the CNV component has revealed significant differences between the AA intervals and the other three interval types, it has not lead to the identification of activity differences that would explain the different intra- vs. intermodal performance levels. However, the analysis performed on the exogenous components revealed that the modulation of N1 and P2 amplitudes depends on whether the sensory stimuli delimiting the intervals were inter- or intramodal. Specifically, the results collected at midline electrodes indicated that the peak of the N1 and P2 components following the presentation of the first stimulus had higher amplitude when the auditory stimulus delimited the beginning of the intermodal intervals (AV) than when it marked the beginning of the intramodal intervals (AA). In the same way, even though the results did not reveal significant differences between intra- and intermodal intervals for the N1 component which appeared after the presentation of the first visual stimuli (VA, VV), they indicated an interaction between the electrode location and the peak amplitude of the visual P2 component. This interaction revealed that the amplitude of the P2 component recorded at Cz and Pz electrodes was higher when the visual signal delimited the beginning of the inter- (VA) compared to the intramodal (VV) intervals. These results suggest that the processing of the intermodal intervals relative to the intramodal interval would differ upon the first 200 milliseconds.

It is usually assumed that the N1-P2 complex reflects stimulus processing that is influenced by selective attention and orientation processes. Different data have revealed that the amplitude of these components was generally higher in attended than unattended conditions [77–79], suggesting that the N1-P2 amplitude may serve as an index of the amount of resources devoted to processing a channel of information. In the same way, the enhancement of these components has been associated with the attentional load and the cognitive effort expended by participants when they have to perform a mentally demanding task [80,81]. Although there are evidences that the characteristics of the N1 and P2 components are influenced by attentional processes, different data also indicated that the amplitude of these components could be modulated by the parameters of presentation of the stimuli. Regarding the latter effect, it has been shown that the presentation of two stimuli of the same sensory modality leads (1) to a decrease of the amplitude of the N1/P2 components associated with the second stimulus [58], and (2) to a rising of the amplitude of the vertex potential (peak-to-peak amplitude of the N1/P2) as a function of the increase of the inter-stimulus interval (ISI) (e.g., [82]). As in the intermodal conditions intervals between stimuli of the same sensory modality from one trial to another were longer than in the intramodal conditions (450 and 550 ms for the short and the long duration, respectively), it could be argued that the larger amplitude of the N1 and P2 components following the first stimuli (S1) for the inter- as compared to intramodal intervals would be a result of these ISI differences. However, even though an effect of the ISI on the amplitude of the components cannot be ruled out, it does not seem to be able to explain by itself the increase of amplitude observed in our study. Indeed, in our study, the duration between S1 (which we analyzed the N1/P2 amplitude) and the previous stimulus of the same modality (S2 for the intramodal condition; S1 for the intermodal condition) was always longer than 4000 ms (including response time), and the difference between intra- vs. intermodal condition was about 450 or 550 ms. Through a meta-analysis combining the results of four groups of researchers who studied the ISI effects on the amplitude of the auditory vertex potential [83], it has been shown that an increase of the ISI in this range is accompanied by a rising of the amplitude which is lower than 6,5%. Given that in our study, the increase in amplitude between the AA and the AV conditions was higher than 15%, we can suppose that other factors, and probably attentional bias, have caused these larger differences. Indeed, in the context of our task, we can assume that the cognitive load was higher when the participants had to process the inter- compared to the intramodal intervals. Contrary to the intramodal intervals, the participants would have to make an attentional switch from one modality to another in order to delimit the beginning and the end of the intermodal intervals. Consequently, the increase of the amplitude of N1 and P2 components may reflect the amount of cognitive resources and particularly, the attentional effort necessary to process the intermodal intervals. The temporal processing being strongly dependent on the efficiency of the attentional mechanisms [84,85], this attentional load may explain the decline in performance observed for intermodal intervals compared to intramodal ones.

## Conclusions

Our behavioural data have confirmed that the temporal performances depended on the sensory modality, performances being higher when the intervals to discriminate were delimited by two auditory rather than two visual signals, and being even more strongly impaired when intermodal intervals are used. Our electrophysiological results have indicated that the activities recorded at fronto-central and parietal sites reflect two distinct mechanisms. The CNV time course recorded at parietal site was independent of the sensory modality and could reflect the implicit establishment of a standard duration. Conversely, the CNV time course recorded at fronto-central site would reflect the explicit processing of the current duration and determine temporal performances. Only the AA intervals induced an increase in amplitude of the CNV over time, suggesting that the high performances obtained in this condition would be related to the neural mechanisms underlying the attentional mechanisms required for the processing of the ongoing duration. Moreover, our data also revealed that the decline in performance observed with intermodal intervals would be related to the contribution of attentional load induced by the necessity to switch from one modality to another.
